# Association between different patterns of social participation and loneliness among the Chinese older people: is there a local-migrant gap?

**DOI:** 10.1186/s12877-024-05391-6

**Published:** 2024-10-01

**Authors:** Rui Chen, Guangwen Liu, Shixue Li, Fanlei Kong

**Affiliations:** 1 Centre for Health Management and Policy Research, School of Public Health, Cheeloo College of Medicine, Shandong University, Jinan, 250012 China; 2 NHC Key Lab of Health Economics and Policy Research, Shandong University, 44 Wenhuaxi Road, Jinan, 250012 China; 3 Institute of Health and Elderly Care, Shandong University, Jinan, 250012 China

**Keywords:** Social participation, Loneliness, Local older people, Migrant older with children

## Abstract

**Background:**

Little empirical evidences were provided on the disparity in the level of loneliness between the migrant older with children (MOC) and their local counterpart in China. This study aimed to explore the association between social participation and loneliness and verify whether there was a local-migrant difference in this association.

**Methods:**

A total of 1332 older people (60 +) were included in this study with 656 MOC and 676 natives. Loneliness was assessed by the University of California Los Angeles Loneliness Scale with eight items (ULS-8). Social participation was evaluated by three kinds of social activities concerning sports activities, hobby activities and community resident interaction. Univariate analysis was conducted to compare the local-migrant disparity as well as the level of loneliness between different subgroups. Hierarchical multiple linear regression analysis was used to examine the proposed relationship and the moderating influence of migration status.

**Results:**

The average ULS-8 scores were 11.73 ± 4.02 for local subjects and 12.82 ± 4.05 for MOC respectively, indicating a lower level of loneliness among local older people. Participating in hobby activities (β = -0.092, *P* = 0.003) and interacting with residents (β = -0.216, *P* = 0.001) more frequently were related to lower level of loneliness while participating in square dancing was related to higher level of loneliness (β = 0.087, *P* = 0.001). The negative relationships between hobby activities as well as resident interaction and loneliness were more profound in migrants than natives.

**Conclusions:**

Only two types of social participation could help alleviate loneliness. More attention to older migrants’ loneliness and extending the scale and types of social activities were recommended for policymakers.

## Background

Population aging, a global phenomenon characterized by the larger and larger proportion of citizens stepping into their later lives, has become a severe social problem in China. The recent National Census showed that the number of older individuals aged 60 years or older was 264 million, accounting for 18.70% of the entire population [1]. According to the projection of National Health Commission of China, the number and proportion of older adults will exceed 400 million and 30% respectively, indicating a severe aging society [2]. Along with the current aging demographics in China, the number of migrant older people was also increasing. A recent report revealed that up to 13.04 million migrant population aged 60 years old or above, comprising 5.9% of all migrants [3]. In addition, Chinese migrant older people shared another feature, that is, most of them moved to urban regions for grandchildren care as well as family reunification [4]. The above two features made the MOC an attracting topic for researchers and a target population for policymakers. In this study, those who leave the familiar rural or urban environment and follow their children to big cities in order to take care of their grandchild or family reunification are referred as the migrant older with children (MOC) [5]. Correspondingly, those who have been living in the household registration area are referred as local older people.

Older migrants were often confronted with a variety of health problems in which loneliness was a noteworthy one [6]. Loneliness is defined as “an adverse emotional response to a perceived disparity between an individual’s desired and actual social relationships” [7]. While lockdown and social isolation measures were being taken to contain the spread of COVID-19, the alarm of a potential “pandemic of loneliness” was raised [8]. A recent meta-analysis of 24 longitudinal studies found a small but significant increase in loneliness since before the outbreak of COVID-19 [9]. Although many previous studies suggested that migrant older people were vulnerable for loneliness due to the narrowing of social circles and changes in lifestyles and living conditions [10, 11], comparatively little empirical evidences were provided on the disparity in the level of loneliness between migrant older people and their local peers. Fokkema et al. compared the level of loneliness between Turkish older migrants and native-born older people in Germany and found that the feelings of loneliness were more prevalent among the Turkish older people than their Germany counterparts [12]. Some Dutch scholars also found that the first-generation migrants were both socially and emotionally lonelier in comparison to their native counterparts [13]. In contrast, the distinction tended to be uncertain in China where intergenerational relationships were traditionally stronger and the adult children were deemed to have the responsibility to provide financial and emotional support on the older adults [14]. On the one hand, considering that the close geographic proximity was beneficial to family interaction and increased the possibility of offspring support in need, Chinese MOC were more likely to experience emotional closeness with children, and thus reported higher level of psychological well-being compared to the local older people [15]. On the other hand, existing studies also documented that Chinese MOC had a significantly higher childcare burden and more pressure from adult children than their local counterparts, which could lead to psychological distress [16]. Therefore, whether the level of loneliness was higher in the Chinese MOC than local older people was still understudied.

Previous studies have revealed a range of factors relevant to geriatric loneliness including sociodemographic features, social resources as well as health and psychological attributes [17]. Among them, social factors were one of the most well-known determinants of loneliness when exploring the local-migrant gaps [18]. Social participation referred to “one’s involvement in activities that offer interaction with others in society or the community” [19]. Few scholars have explored the association between social participation and loneliness among Chinese older migrants. Pan et al.'s study of loneliness among Chinese migrants during the pandemic found that reduced social participation led to increased loneliness [20]. The association between social participation and loneliness has been explored primarily in local older adults. A recent study conducted in Iran revealed that social participation barriers were one of the best predictors of loneliness among the community-dwelling older people [21]. Another qualitative research among the lonely older adults living in London also concluded that addressing the fears of social rejection and losing social identities could enhance social participation, and further alleviate their level of loneliness [22]. A meta-analysis on randomized comparison studies found that interventions on social participation had a significant effect on loneliness reduction despite that the effect size was small [23]. Moreover, a positive relationship between lower level of social participation and loneliness was found among the community-dwelling older people in Anhui, China [24]. Another study of older people across China also revealed that older people who were more actively involved in activities were more likely to be less lonely [25]. Nevertheless, although previous studies indicated a limited community participation of older migrants [26], fewer scholars paid attention to the correlation between social participation and loneliness among the Chinese MOC, let alone the comparison between locals and migrants.

The loneliness model raised by de Jong-Gierveld in 1987 offered explanation on the association between social participation and loneliness as well as the probable local-migrant disparity.According to her theoretical model, both the subjective evaluation and the descriptive features of the social network were directly related to the experience of loneliness. Additionally, the geographic mobility was one of the background variables which served as possible moderators [27]. Another theory proposed by Berkman et al. stated that social participation could affect health status through psychological path including depression, distress and other adverse mental health outcomes. She also pointed out that social structural conditions such as public policy, culture and socioeconomic status were upstream factors of the social participation [28]. Considering the inequality in those social structural factors among two groups of participants in the current study, it is plausible that the impact of social participation on health might differ between the MOC and local older individuals.

Existing articles in the authors’ team had explored the prevalence and determinants of loneliness of MOC, and found that oral health [29], sense of belonging [30], social support and smartphone usage [31], acculturation and family support [32] were correlated with loneliness. However, these studies only focused on the MOC as the target population, did not compare the MOC with the local older people and never explored the association between social participation and loneliness. Thus, this study aimed to explore the prevalence of loneliness and its determinants from the perspective of the local older people-migrant older people comparison.

To summarize, no study had investigated social participation and loneliness among the local older adults and MOC simultaneously in China. Thus, this current study aimed to compare the level of loneliness between native older people and the MOC, explore the association between social participation and loneliness and examine the local-migrant disparity of this relationship. There are three hypotheses. Firstly, there is significant difference in the level of loneliness between the local older people and MOC, with a higher level of loneliness among the MOC than local older people. Secondly, social participation is significantly and negatively associated with loneliness among the native older people as well as the migrant counterparts. Thirdly, the relationship between social participation and loneliness among the older adults differs by their migration status, with stronger correlations among the MOC than local older people.

## Methods

### Data collection and the research subjects

The data was collected in Jinan City, Shandong Province, China in August 2020.The gross domestic product of Shandong Province was 9.3 trillion Chinese Yuan (≈1.3 trillion US$) in 2021 [33]. The total population of Shandong Province was 101 million by the end of 2020 according to the Seventh National Census [34]. Jinan City is the capital of Shandong Province, one of the Chinese eastern provinces. The gross domestic product of Jinan in 2020 was 1.01 trillion Chinese Yuan (≈157,285.51 million US$) [35]. Jinan governed 10 districts and 2 counties (132 sub-districts and 29 towns) until July 2020 [36]. As of November 2020, Jinan had a total of 9.20 million local residents [37], while the number of registered population was 8.06 million [38]. In 2020, nearly 35.83% (3.29 million) of its whole population constituted migrants from other counties, cities or provinces with a variety of sociodemographic and cultural backgrounds. Thus, two groups of older people who aged 60 years or above were recruited in this study. Migrant older people were defined as those who followed their children to Jinan City whereas local older people were defined as those who were currently living and had their household registered in the study area.

To choose both the local older people and MOC, multi-stage cluster random sampling was conducted. In the first stage, three of ten districts were selected as the primary sampling units (PSUs), considering the economic development and the geographic location. In the second stage, a total of three sub-districts were chosen as the secondary sampling units (SSUs) from each PSU; that is, one sub-district was selected from each of the districts chosen previously. In the last stage, three communities were chosen from the SSUs; that is, one community was selected from each of the sub-districts chosen previously. All the MOC as well as the local older people who met the above criteria constituted the total study sample. The inclusion criteria for the participants were (1) age 60 years and above and (2) clear awareness and cognition. The MOC’s household registration was beyond Jinan City, while the local older people’s household registration was in Jinan City.

Thirty-two college students were recruited as investigators after the training about the background of the whole study, questionnaire content, and the technique on social survey. Twenty-minute face-to-face data collection processes were conducted between the investigators and subjects. Some of the interviews were held in participants’ home after their permission while others were held in public areas of the communities. Before every interview, the consent to participate were obtained by asking the respondents whether they had time and were willing to join the survey after the introduction of the background and the purpose of the research.

At first, a total of 670 MOC as well as 686 local older people were selected and interviewed. However, 14 of the MOC and 10 of the locals were removed from the sample because of obvious logical errors or uncompleted questionnaires, resulting in a valid response rate of 98.23%. Finally, 1332 older adults were included in the database, where 656 MOC and 676 local elders were thus analyzed.

### Measurements

#### Dependent variable

Loneliness was assessed by the Chinese version of eight-item University of California Los Angeles Loneliness Scale (ULS-8). Russell firstly invented the initial 20-item University of California Los Angeles (UCLA) Loneliness Scale and revised it to reduce the response bias [7, 39]. Hays and DiMatteo then chose eight items on the basis of the revised UCLA Loneliness scale and designed ULS-8 [40]. Some Chinese scholars have translated it and proved a good reliability and validity [41, 42]. Each item was evaluated on a 4-point Likert scale ranging from 1 (“never”) to 4 (“always”) and thus a total score of 8 to 32 was generated. Higher scores indicated higher level of loneliness. The Cronbach’s α value for the scale was 0.82.

#### Independent variables

##### Social participation

Social participation was measured by three kinds of social activities which were used in the China Health and Retirement Longitudinal Survey and other studies [43, 44]. Older individuals were asked the following questions: (a) “How often did you participate in sports activities such as square dancing?”, (b) “How often did you participate in hobby activities?” and (c) “How often did you interact with community residents?”. They could select one answer from the frequency of “Never”, “Seldom”, “Sometimes” and “Often”.

##### Migration status

Migration status was evaluated by asking the older adults “Where did you register your household?”. Those who followed their children to the study area and did not register their household here were defined as MOC. Those who were currently living and had their household registered in the study area were defined as local older people.

##### Covariables

Sociodemographic variables and health status of the participants were included in the current study as covariates because previous researchers had recognized them as main risk factors, especially in the comparison studies between local and migrants [45, 46]. Age was divided into 60–69 years old, 70–79 years old and 80 year or above; gender was categorized as male and female; marital status was divided into currently married or single (such as divorced, widowed, unmarried, etc.); educational level was coded as illiterate, primary school or middle school or above; religious belief was coded as no or yes; household monthly income was divided into four categories according to the quartile; source of living expenses was classified into own pension, own savings, from others and basic living allowances. With respect to the health status, older individuals were asked about the hearing status and chronic disease. Response to the former was normal or impaired and response to the latter was yes or no. Furthermore, the Short-Form Health Survey (SF-12) was also used to measure their physical and mental health [47]. SF-12 scores were divided into physical component summary (PCS) and mental component summary (MCS) scores [48] and both of them could be dichotomized by the cut-off point of the first quartile [49]. Following a previous study did, participants whose PCS/MCS scores were lower than the first quartile were defined as poor physical/mental health while those whose scores were higher than the first quartile were defined as good physical/mental health [50].

### Statistical analysis

Data were presented as the mean and standard deviation for continuous variables, while frequency and percentage for categorical variables. T-test and Chi square test were used to compare the basic features and health status among the local and migrant older people. Univariate analysis (including t-test and one-way ANOVA) was conducted to compare the difference in ULS-8 scores among the different subgroups of sociodemographic variables, health status and social participation indicators. Those sociodemographic variables and health status indicators which were significantly correlated with loneliness were introduced into further analysis. Hierarchical multiple linear regression analysis was then conducted to estimate independent associations between each type of social participation and loneliness as well as the heterogeneity of the migration status. It contained ten models in total. Firstly, Model 1 to Model 3 included each indicator of social participation along with the migration status variable. Sociodemographic characteristics and health status were then brought into each Model 1 to Model 3 as controlling variables. Next, all the social participation and migration variables were entered in Model 7 along with all the covariates and the association between social participation and loneliness was finally determined. The interaction terms (social participation*migration status) were further added to Model 8 to Model 10 to test the moderating influence of migration status on this association. Margins plots were also employed to clearly illustrated the significant interaction of migration status and different kinds of social participation. Statistical differences were considered significant when *p* ≤ 0.05. All analyses were performed using Stata 14.2.

### Ethical considerations

Medical ethics approval of this study was approved by the Ethical Committee of School of Public Health, Shandong University (No. 20180225). Informed consent for the data collection and the use of the data was obtained from all subjects.

## Results

### Basic characteristics among the older people and the local-migrant disparities

Table 1 depicts the fundamental features of the participants. A total of 1332 older people with the average age of 67.80 ± 7.03 were included in the current research. Among them, 50.75% were native older people and 49.25% were MOC. The mean ULS-8 score was 12.27 ± 4.07 among the older individuals. Respondents were predominantly 60 to 69 years old (69.06%), female (63.14%), currently married (82.66%), received a middle school or above education level (56.98%), had no religious belief (95.50%) and lived on their own pension (48.95%). Considering the health status, approximately half of them did not develop chronic disease and more than 80% reported normal hearing status. Nearly three quarters reported good physical health as well as mental health, which were represented by the corresponding two components of SF-12. As for the social participation indicators, 1012 older adults never participated in sports activities (such as square dancing), accounting for 75.98% of the total. Over one third of them never participated in hobby activities, followed by seldom (336, 25.23%) and often (297, 22.30%). In contrast, 58.18% of the whole respondents often interacted with community residents and the proportion of those who never interacted with community residents was merely 5.10%.

**Table 1:** The comparison of sociodemographic features, social participation indicators and loneliness between local and migrant older people in Jinan, China

Variables	Total (%)	Local older people (%)	Migrant older people (%)	${\chi }^{2}$/t	*P*
Observation	1332 (100.00)	676 (50.75)	656 (49.25)	-	-
Age (year)				147.553^a^	** < 0.001**
60–69	920 (69.06)	368 (54.44)	552 (84.15)		
70–79	300 (22.52)	210 (31.07)	90 (13.72)		
80 or above	112 (8.40)	98 (14.50)	14 (2.13)		
Gender				0.188^a^	0.665
Male	494 (37.08)	253 (37.43)	238 (36.28)		
Female	841 (63.14)	423 (62.57)	418 (63.72)		
Marital status				34.882^a^	** < 0.001**
Currently married	1101 (82.66)	518 (76.63)	583 (88.87)		
Single^c^	231 (17.34)	158 (23.37)	73 (11.13)		
Educational level				65.850^a^	** < 0.001**
Illiterate	280 (21.02)	84 (12.43)	196 (29.88)		
Primary school	293 (22.00)	149 (22.04)	144 (21.95)		
Middle school or above	759 (56.98)	443 (65.53)	316 (48.17)		
Religious belief				3.876^a^	**0.049**
No	1272 (95.50)	653 (96.60)	619 (94.36)		
Yes	60 (4.50)	23 (3.40)	37 (5.64)		
Household monthly income^d^				269.367^a^	** < 0.001**
Q1	348 (26.13)	109 (16.12)	239 (36.43)		
Q2	326 (24.47)	86 (12.72)	240 (36.59)		
Q3	331 (24.85)	224 (33.14)	107 (16.31)		
Q4	327 (24.55)	257 (38.02)	70 (10.67)		
Source of living expenses				207.319^a^	** < 0.001**
Own pension	652 (48.95)	458 (67.75)	194 (29.57)		
Own savings	83 (6.23)	42 (6.21)	41 (6.25)		
From others	549 (41.22)	163 (24.11)	386 (58.84)		
Basic living allowances	48 (3.60)	13 (1.92)	35 (5.33)		
Chronic disease				13.527^a^	** < 0.001**
No	673 (50.53)	308 (45.56)	365 (55.64)		
Yes	659 (49.47)	368 (54.44)	291 (44.36)		
Hearing status				18.273^a^	** < 0.001**
Normal	1122 (84.23)	541 (80.03)	581 (88.57)		
Impaired	210 (15.77)	135 (19.97)	75 (11.43)		
Physical health (PCS)				9.227^a^	**0.002**
Poor	333 (25.00)	193 (28.55)	140 (21.34)		
Good	999 (75.00)	483 (71.45)	516 (78.66)		
Mental health (MCS)				0.785^a^	0.376
Poor	331 (24.85)	161 (23.82)	170 (25.91)		
Good	1001 (75.15)	515 (76.18)	486 (74.09)		
Participating in sports activities like square dancing				9.338^a^	**0.025**
Never	1012 (75.98)	520 (76.92)	492 (75.00)		
Seldom	123 (9.23)	48 (7.10)	75 (11.43)		
Sometimes	74 (5.56)	37 (5.47)	37 (5.64)		
Often	123 (9.23)	71 (10.50)	52 (7.93)		
Participating in hobby activities				34.344^a^	** < 0.001**
Never	459 (34.46)	209 (30.92)	250 (38.10)		
Seldom	336 (25.23)	146 (21.60)	190 (28.96)		
Sometimes	240 (18.02)	131 (19.38)	109 (16.62)		
Often	297 (22.30)	190 (28.11)	107 (16.31)		
Interaction with community residents				19.031^a^	** < 0.001**
Never	68 (5.10)	30 (4.44)	38 (5.79)		
Seldom	158 (11.86)	73 (10.80)	85 (12.96)		
Sometimes	331 (24.85)	141 (20.86)	190 (28.96)		
Often	775 (58.18)	432 (63.90)	343 (52.29)		
Loneliness, mean ± SD	12.27 ± 4.07	11.73 ± 4.02	12.82 ± 4.05	-4.935^b^	** < 0.001**

*PCS* Physical component summary, *MCS* Mental component summary, *SD* Standard deviation

^a^Chi-square

^b^T value

^c^Single included those who were unmarried (5, 0.37%), divorced (9, 0.67%), widowed (207, 15.54%) and under other circumstances (10, 0.75%)

^d^Q1 was the poorest and Q4 was the richest

In addition, significant differences in almost all the research variables were found between the local older people and MOC, except for the gender and mental health. In detail, the MOC tended to be younger, currently married and have a lower educational level compared to the local older people (*P* < 0.001). Most of the MOC belonged to the families with monthly income in the lowest quartile and got living expenses from others (such as adult children and sibling) while the majority of their local counterpart belonged to the families with highest income and lived on their own pension (*P* < 0.001). With respect to the health status, local older people were more likely to suffer from chronic disease (*P* < 0.001), hearing impairment (*P* < 0.001), and poor physical health (*P* = 0.002) compared with the MOC. Regarding the comparison of social participation and loneliness between the above two groups, migrant older people had a higher average score in ULS-8 than local older people (*P* < 0.001), indicating that the MOC was lonelier than their local counterpart. It was also found that local participants were more likely to take part in sports activities (*P* = 0.025), hobby activities (*P* < 0.001) and interact with community residents (*P* < 0.001) with a higher frequency.

### Association between social participation and loneliness

As can be seen in Table 2, there were significant differences in the mean score of ULS-8 among different subgroups of three social participation indicators according to the results of univariate analysis. Respondents who sometimes engaged in the square dancing (*P* = 0.002), seldom participated in hobby groups (*P* < 0.001) and never contacted with community residents (*P* < 0.001) had a higher score of loneliness. These relationships were further examined by the hierarchical multiple regression analysis, which was showed in Table 3. Models 1 to Model 3 introduced each type of social participation and migration status variables, respectively. Model 1 verified that there was a positive association between sports activities and loneliness after controlling for migration status. Models 2 and Model 3 clarified the significantly negative association between hobby activities and loneliness as well as resident interaction and loneliness after controlling for migration status. Models 4 to Model 6 added the sociodemographic variables (including age, education level, household monthly income and source of living expenses) and health status indicators (including physical and mental health) that were found to be significantly correlated with loneliness in the univariate analysis, respectively. It was revealed that the relationship between each social participation variable and loneliness remained significant after the inclusion of the covariates, but the estimates of the correlation coefficients decreased. Model 7 then included the three social participation indicators as well as socio-demographic variables and health status indicators in the model. The results showed that the relationship between sports activities and loneliness remained significantly positive, and the relationship between hobby activities and loneliness as well as resident interaction and loneliness remained significantly negative, but the coefficient of hobby activities with the frequency of often became insignificant. In addition, the estimates for physical activity and hobbies increased while the estimates for resident contact decreased. Specifically, in the fully adjusted model, participating in square dancing was positively associated with loneliness (β for seldom = 0.094, β for sometimes = 0.087, *P* = 0.001), meaning that older adults who engaged in square dancing with the frequency of seldom and sometimes reported an average increase of 0.094 and 0.087 points in the score of ULS-8 respectively, compared with those who never took part in square dancing. However, participating in hobby activities was negatively related to loneliness (β = -0.092, *P* = 0.003), indicating that those who sometimes attended hobby activities had an average decrease of 0.092 in the ULS-8 compared with their non-participation counterparts. Finally, those who interacted with community residents had a lower level of loneliness in comparison with those who did not (β for seldom = -0.103, *P* = 0.024; β for sometimes = -0.110, *P* = 0.049; β for often = -0.216, *P* = 0.001).

**Table 2:** Univariate analysis on the association between covariables, social participation and loneliness among the older adults in Jinan, China

Variables	N (%)	Mean score of ULS-8 (SD)	t /F value	*P*
Observation	1332 (100.00)	12.27 (4.07)	-	-
Age (year)			3.692^a^	**0.025**
60–69	920 (69.06)	12.30 (3.98)		
70–79	300 (22.52)	11.87 (4.01)		
80 or above	112 (8.40)	13.07 (4.81)		
Gender			-0.186^b^	0.852
Male	494 (37.08)	12.24 (4.06)		
Female	841 (63.14)	12.29 (4.07)		
Marital status			-1.170^b^	0.242
Currently married	1101 (82.66)	12.21 (3.94)		
Single^c^	231 (17.34)	12.55 (4.63)		
Educational level			6.367^a^	**0.002**
Illiterate	280 (21.02)	12.88 (4.24)		
Primary school	293 (22.00)	12.54 (4.27)		
Middle school or above	759 (56.98)	11.94 (4.14)		
Religious belief			1.272^b^	0.204
No	1272 (95.50)	12.30 (4.09)		
Yes	60 (4.50)	11.62 (3.63)		
Household monthly income^d^			4.369^a^	**0.005**
Q1	348 (26.13)	12.61 (4.26)		
Q2	326 (24.47)	12.70 (3.92)		
Q3	331 (24.85)	12.00 (4.21)		
Q4	327 (24.55)	11.75 (3.79)		
Source of living expenses			2.872^a^	**0.035**
Own pension	652 (48.95)	12.19 (4.24)		
Own savings	83 (6.23)	11.93 (2.61)		
From others	549 (41.22)	12.54 (4.06)		
Basic living allowances	48 (3.60)	10.92 (3.51)		
Chronic disease			0.722^b^	0.470
No	673 (50.53)	12.35 (3.80)		
Yes	659 (49.47)	12.19 (4.32)		
Hearing status			0.140^b^	0.888
Normal	1122 (84.23)	12.28 (4.03)		
Impaired	210 (15.77)	12.23 (4.30)		
Physical health (PCS)			3.125^b^	**0.002**
Poor	333 (25.00)	12.87 (4.31)		
Good	999 (75.00)	12.07 (3.97)		
Mental health (MCS)			2.856^b^	**0.004**
Poor	331 (24.85)	12.82 (4.20)		
Good	1001 (75.15)	12.09 (4.01)		
Participating in sports activities like square dancing			5.084^a^	**0.002**
Never	1012 (75.98)	12.17 (4.06)		
Seldom	123 (9.23)	13.23 (4.52)		
Sometimes	74 (5.56)	13.20 (4.17)		
Often	123 (9.23)	11.55 (3.29)		
Participating in hobby activities			7.445^a^	** < 0.001**
Never	459 (34.46)	12.59 (4.38)		
Seldom	336 (25.23)	12.82 (4.20)		
Sometimes	240 (18.02)	11.72 (3.66)		
Often	297 (22.30)	11.58 (3.58)		
Interaction with community residents			7.080^a^	** < 0.001**
Never	68 (5.10)	13.60 (4.82)		
Seldom	158 (11.86)	12.43 (4.04)		
Sometimes	331 (24.85)	12.83 (4.30)		
Often	775 (58.18)	11.88 (3.85)		

*SD* Standard deviation, *PCS* Physical component summary, *MCS* Mental component summary

^a^F value

^b^T value

^c^Single included those who were unmarried (5, 0.37%), divorced (9, 0.67%), widowed (207, 15.54%) and under other circumstances (10, 0.75%)

^d^Q1 was the poorest and Q4 was the richest

**Table 3:** Hierarchical multiple regression analysis on the association between social participation and loneliness among the older adults in Jinan, China

Variables	Model 1			Model 2		Model 3		Model 4		Model 5		Model 6		Model 7	
	β (95% CI)		*P*-value	β (95% CI)	*P*-value	β (95% CI)	*P*-value	β (95% CI)	*P*- value	β (95% CI)	*P*- value	β (95% CI)	*P*- value	β (95% CI)	*P*- value
Participating in sports activities like square dancing															
Never	Ref.							Ref.						Ref.	
Seldom	0.069 (0.232, 1.774)		**0.011**					0.075 (0.324, 1.856)	**0.005**					0.094 (0.595, 2.148)	**0.001**
Sometimes	0.067 (0.261, 2.201)		**0.013**					0.063 (0.200, 2.124)	**0.018**					0.087 (0.623, 2.572)	**0.001**
Often	-0.025 (-1.137, 0.402)		0.349					-0.013 (-0.948, 0.584)	0.641					0.027 (-0.446, 1.236)	0.357
Participating in hobby activities															
Never				Ref.						Ref.				Ref.	
Seldom				0.023 (-0.353, 0.801)	0.446					0.015 (-0.428, 0.718)	0.619			0.006 (-0.527, 0.649)	0.839
Sometimes				-0.094 (-1.667, -0.384)	**0.002**					-0.082 (-1.533, -0.258)	**0.006**			-0.092 (-1.665, -0.355)	**0.003**
Often				-0.081 (-1.430, -0.223)	**0.007**					-0.068 (-1.290, -0.088)	**0.025**			-0.062 (-1.312, 0.069)	0.078
Interaction with community residents															
Never						Ref.						Ref.		Ref.	
Seldom						-0.106 (-2.547, -0.217)	**0.020**					-0.104 (-2.502, -0.194)	**0.022**	-0.103 (-2.496, -0.178)	**0.024**
Sometimes						-0.117 (-2.204, -0.065)	**0.038**					-0.121 (-2.234, -0.115)	**0.030**	-0.110 (-2.145, -0.002)	**0.049**
Often						-0.230 (-2.977, -0.943)	**<0.001**					-0.224 (-2.921, -0.905)	**<0.001**	-0.216 (-2.879, -0.805)	**0.001**
Migration status															
Local	Ref.			Ref.		Ref.		Ref.		Ref.		Ref.		Ref.	
Migrant	0.193 (1.181, 2.067)		**<0.001**	0.182 (1.090, 1.983)	**<0.001**	0.187 (1.130, 2.017)	**<0.001**	0.204 (1.188, 2.244)	**<0.001**	0.196 (1.111, 2.178)	**<0.001**	0.199 (1.148, 2.204)	**<0.001**	0.176 (0.951, 2.014)	**<0.001**
Age (year)															
60-69								Ref.		Ref.		Ref.		Ref.	
70-79								-0.007 (-0.637, 0.495)	0.806	-0.009 (-0.652, 0.480)	0.765	-0.016 (-0.721, 0.409)	0.588	-0.012 (-0.683, 0.441)	0.674
80 or above								0.064 (0.099, 1.825)	**0.029**	0.056 (-0.017, 1.712)	0.055	0.062 (0.080, 1.800)	**0.032**	0.059 (0.039, 1.751)	**0.040**
Educational level															
Illiterate								Ref.		Ref.		Ref.		Ref.	
Primary school								-0.009 (-0.767, 0.495)	0.780	-0.011 (-0.787, 0.566)	0.749	-0.010 (-0.773, 0.582)	0.782	-0.011 (-0.782, 0.562)	0.748
Middle school or above								-0.054 (-1.078, 0.155)	0.142	-0.058 (-1.111, 0.120)	0.115	-0.060 (-1.127, 0.103)	0.102	-0.049 (-1.029, 0.193)	0.179
Household monthly income^a^															
Q1								Ref.		Ref.		Ref.		Ref.	
Q2								-0.014 (-0.767, 0.495)	0.673	-0.015 (-0.776, 0.485)	0.651	-0.015 (-0.778, 0.480)	0.643	-0.018 (-.796, 0.453)	0.590
Q3								-0.066 (-1.340, 0.060)	0.073	-0.060 (-1.284, 0.115)	0.101	-0.059 (-1.272, 0.123)	0.106	-0.067 (-1.347, 0.040)	0.065
Q4								-0.075 (-1.518, 0.527)	0.067	-0.064 (-1.411, 0.154)	0.115	-0.063 (-1.398, 0.165)	0.122	-0.073 (-1.496, 0.060)	0.071
Source of living expenses															
Own pension								Ref.		Ref.		Ref.		Ref.	
Own savings								-0.037 (-1.599, 0.314)	0.188	-0.029 (-1.463, 0.451)	0.300	-0.028 (-1.460, 0.453)	0.302	-0.029 (-1.456, 0.442)	0.295
Others								-0.073 (-1.213, -0.032)	**0.039**	-0.065 (-1.148, 0.035)	0.065	-0.069 (-1.176, 0.003)	0.051	-0.065 (-1.141, 0.034)	0.065
Basic living allowances								-0.107 (-3.656, -1.166)	**<0.001**	-0.104 (-3.583, -1.095)	**<0.001**	-0.109 (-3.688, -1.207)	**<0.001**	-0.100 (-3.500, -1.032)	**<0.001**
Physical health (PCS)															
Poor								Ref.		Ref.		Ref.		Ref.	
Good								-0.076 (-1.269, -0.203)	**0.007**	-0.073 (-1.243, -0.178)	**0.009**	-0.071 (-1.219, -0.155)	**0.011**	-0.071 (-1.213, -0.158)	**0.011**
Mental health (MCS)															
Poor								Ref.		Ref.		Ref.		Ref.	
Good								-0.063 (-1.123, -0.094)	**0.020**	-0.055 (-1.050, -0.019)	**0.042**	-0.060 (-1.099, -0.072)	**0.025**	-0.055 (-1.047, -0.027)	**0.039**
F	17.339		**<0.001**	18.991	**<0.001**	19.215	**<0.001**	7.384	**<0.001**	7.426	**<0.001**	7.777	**<0.001**	7.114	**<0.001**
$R_c^2$	0.047			0.051		0.052		0.071		0.072		0.075		0.092	
$\triangle R_c^2$	-			0.004		0.001		0.019		0.001		0.003		0.017	

Note: β: Standardized coefficients, PCS: Physical component summary, MCS, Mental component summary

a: Q1 was the poorest and Q4 was the richest

### Interaction between migration status and social participation on loneliness

**Fig. 1:**
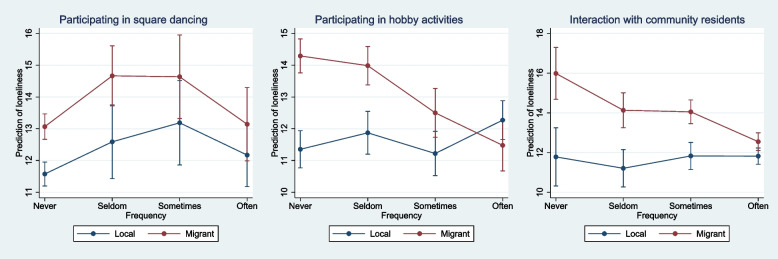
Margins plot of the interaction between social participation and migration status in the prediction of loneliness

Table 4 illustrates the moderating influence of migration status on the association between social participation and loneliness. Interaction terms between migration status and different kinds of social participation were added into Model 8 to Model 10 respectively to test the heterogeneity of migration status. In Model 8, the interplay between migration status and the participation in sports activities was insignificant, revealing that the strength of relationship between sports activities participation and loneliness did not differ in the local older people and MOC. On the contrary, the MOC who participated in hobby activities and interacted with community residents were significantly and negatively correlated with ULS-8 score (β for sometimes*migrant = -0.108, *P* = 0.010; β for often*migrant = -0.241, *P* < 0.001, in Model 9; β for often*migrant = -0.362, *P* = 0.001, in Model 10). Figure 1 provided clear evidence on the local-migrant discrepancy by calculating and comparing the slopes of migration status on different frequencies of social participation activities. It was found that the association between hobby activities with the frequency of sometimes and often and loneliness was pronounced for the MOC than local counterpart. Similarly, the relationship between resident interaction with the frequency of often and loneliness was distinct in the MOC than local participants.

**Table 4:** The interaction between migration and social participation on loneliness among the older adults in Jinan, China

Variables	Model 8			Model 9		Model 10	
	β (95% CI)	*P*-value		β (95% CI)	*P*-value	β (95% CI)	*P*-value
Migration status							
Local	Ref.						
Migrant	0.177 (0.904, 2.080)	**<0.001**		0.350 (2.130, 3.750)	**<0.001**	0.500 (2.250, 6.163)	**<0.001**
Participating in sports activities like square dancing							
Never	Ref.			Ref.		Ref.	
Seldom	0.070 (-0.189, 2.217)	0.098		0.090 (0.529, 2.071)	**0.001**	0.096 (0.623, 2.166)	**<0.001**
Sometimes	0.088 (0.249, 2.976)	**0.021**		0.083 (0.559, 2.490)	**0.002**	0.083 (0.550, 2.486)	**0.002**
Often	0.041 (-0.458, 1.652)	0.267		0.040 (-0.254, 1.411)	0.173	0.034 (-0.346, 1.325)	0.251
Participating in hobby activities							
Never	Ref.			Ref.		Ref.	
Seldom	0.006 (-0.534, 0.647)		0.852	0.054 (-0.332, 1.369)	0.232	0.002 (-0.561, 0.607)	0.939
Sometimes	-0.092 (-1.666, -0.352)		**0.003**	-0.012 (-1.017, 0.751)	0.767	-0.009 (-1.667, -0.366)	**0.002**
Often	-0.059 (-1.290, 0.097)		0.092	0.091 (0.068, 1.770)	**0.034**	-0.007 (-1.381, -0.007)	**0.048**
Interaction with community residents							
Never	Ref.			Ref.		Ref.	
Seldom	-0.103 (-2.500, -0.179)		**0.024**	0.102 (-2.469, -0.177)	**0.024**	-0.004 (-2.283, 1.135)	0.510
Sometimes	-0.110 (-2.140, 0.004)		0.051	-0.109 (-2.115, -0.001)	**0.050**	0.005 (-1.545, 1.640)	0.953
Often	-0.251 (-2.874, -0.798)		**0.001**	-0.209 (-2.807, -0.759)	**0.001**	0.004 (-1.474, 1.550)	0.960
Participating in sports activities * migration status							
Never * migrant	Ref.						
Seldom* migrant	0.032 (-0.971, 2.141)		0.461				
Sometimes * migrant	-0.002 (-1.958, 1.874)		0.966				
Often * migrant	-0.024 (-2.055, 1.013)		0.505				
Participating in hobby activities* migration status							
Never * migrant				Ref.			
Seldom * migrant				-0.069 (-1.959, 0.309)	0.154		
Sometimes * migrant				-0.108 (-2.917, -0.401)	**0.010**		
Often * migrant				-0.241 (-4.950, -2.518)	**<0.001**		
Interaction with community residents * migration status							
Never * migrant						Ref.	
Seldom * migrant						-0.007 (-3.570, 1.008)	0.272
Sometimes * migrant						-0.165 (-4.090, 0.126)	0.065
Often * migrant						-0.362 (-5.478, -1.475)	**0.001**
F	6.296		**<0.001**	7.942	**<0.001**	7.238	**<0.001**
$R^{2}_{c}$	0.090			0.115		0.105	
$\Delta R^{2}_{c}$	-0.002			0.023		0.013	

All models adjusted for age, educational level, household monthly income, source of living expenses, physical health and mental health among the participants. β: Standardized regression coefficients

## Discussion

To the best knowledge, this was the first study that examined the local-migrant gap in loneliness as well as the impact of social participation on it among the Chinese older individuals. It was found that Chinese MOC felt lonelier compared to the local counterpart. The hypothesized negative association between social participation and loneliness was merely verified in two types of social activities whereas the above relationship was found to be positive in the remaining one. Likewise, the hypothesized difference in social participation-loneliness link between local older people and migrant older people was also observed only in two activity types.

### The level of loneliness among the local older people and MOC in Jinan, China

The mean scores of ULS-8 were 12.82 ± 4.05 and 11.73 ± 4.02 in the migrant participants and local respondents respectively, suggesting that the level of loneliness among the migrant older people was higher. It was consistent with a Canadian research which revealed that the level of loneliness among the immigrants from non-European countries was higher compared to the native-born older people [51]. Another study also demonstrated that older migrants from a non-English-speaking country in Australia were lonelier in comparison with the Australian-born older individuals after adjusting neighborhood characteristics and sociodemographic variables [52]. A study conducted in Shanghai also found a stronger sense of loneliness among migrants than local urban residents despite that the study participation was young and middle-aged adults [53]. The possible explanation may due to the MOC face many adaptation problems when they migrated to a new place. For example, they may experience cultural adjustment stress [54]. Specifically for the current study, it was noted that most of the MOC moved from rural areas [55]. The attachment to the farming land among the rural older people made it difficult for old couples to migrate to urban areas together, thus leading to higher level of loneliness [56]. In addition, after migration, the MOC's original support network may break down and it is difficult to form a new urban support network within a short period of time [57], which may also lead to higher levels of loneliness for the MOC.

### The relationship between social participation and loneliness

Results in the current study showed a significant association between three kinds of social participation and loneliness although the direction of these relationships was different. Participating in hobby activities and interacting with community residents with a higher frequency were related to the alleviation of loneliness, which was in line with previous studies. Former evidence indicated that more frequent leisure activities predicted better mental well-being, including lower depressive symptoms, anxiety and loneliness [58]. The homebound older people with self-reported loneliness were less likely to engage in several meaningful activities, especially the leisure ones [59]. As the essential part of social participation [60], interaction with community residents could provide social support for the older people, and thus mitigated their level of loneliness to a large degree [61]. However, the positive impact of participating in sports activities on loneliness was inconsistent with former studies because most previous scholars believed that exercise and sports could delay the decline in seniors’ activities of daily living, maintain the cognitive function and finally avoid the loneliness[62, 63]. The reason for this may be that in China, square dancing is not only a recreational activity, but also frequently appears in public life as a competition activity [64]. Square dancing participants might be confronted with great pressure for the fear of not being able to help the team win the competition, particularly for those who seldom or sometimes attended this kind of sports activity and were unfamiliar with dance movements. Therefore, the older people who seldom and sometimes attended square dancing reported a lower self-evaluation and thus a higher loneliness compared to the non-participants [65]. Furthermore, given the social context of the COVID-19 pandemic at that time, older people might experience loneliness due to the unfulfilled desire of doing sport activities (e.g. restrictive measures, or fear of being infected if they go out). In this sense, the older people who seldom and sometimes participated in square dancing might have a higher level of loneliness.

### The heterogeneity of migration status in the social participation and loneliness relationship

It was noteworthy that the correlation between hobby activities participation and loneliness was more profound in the MOC than local older people. For one hand, attending hobby activities was a crucial way to make new friends for those migrating from rural areas, considering that they lost contact with almost all old fellows in origin places [11]. Comparatively, hobby activities in local older people were just one way for personal amusement. For another hand, due to the distinction in the economic development between urban and rural in China [66], rural MOC were more likely to find new leisure activities which the local older people have been accustomed to. The above two reasons made it easier for the MOC who participated in hobby activities and contacted community residents with a higher frequency to mitigate loneliness compared to the local counterparts. As for the association between sports activities like square dancing and loneliness, no heterogeneity was found between migrants and locals mainly because the participation rate of square dancing in the sample communities was relatively low (no more than 25% in this study while more than 50% in the former one [44]), indicating a limit impact on loneliness.

### Implications

Our current study would provide following implications. Firstly, the MOC in urban areas should be deemed as a key population by the municipal civil affairs department when addressing the geriatric mental health problems. Secondly, policymakers ought to take measures to enhance the social participation of older adults such as extending the scale and types of social activities by building multifunctional activity rooms and constructing age-friendly communities. Thirdly, given that square dancing participation exerted a positive influence on loneliness and the influence did not vary by migration status, the government are supposed to provide suitable fields and decrease the number of competitions in square dancing to encourage more older people to enjoy it.

### Limitations

There are also some limitations. Firstly, the cross-sectional design brought about hindrance in inferring the causal relationship. Secondly, there was no universally accepted scale on assessing social participation currently, while some existed scale could not cover all kinds of social participation activities in different cultural background regions, thus the questions used to assess social participation in this study needed further improvement. Thirdly, the frequency of social participation was self-reported data, which made recall and reporting bias inevitable. Fourthly, due to the pandemic of COVID-19, this study only completed the questionnaire survey in Jinan. In the future, surveys should be conducted in other areas to extend the generalizability. Fifthly, although the economic development and geographical variation was taken into consideration during the sampling stage of this study, but it was not used for the data analysis. Lastly, as the study was conducted during the COVID-19 pandemic times, there might be potential effect of the pandemic on social participation and loneliness.

## Conclusions

The level of loneliness among the MOC was higher than that reported among the local older people in Jinan, China. Social participation pertaining to hobby activities and resident interaction was found to be negatively associated with loneliness Furthermore, we also found that this negative association varied by migration status, with a more significant negative association between hobby activities as well as resident interaction and loneliness compared to the local older people. More attention to older migrants’ loneliness and extending the scale and types of social activities are recommended for policymakers. Moreover, the above findings of this study should be interpreted with caution when considering their application to non-COVID times, since the data collection was conducted during the COVID-19 pandemic (August, 2020).

## Data Availability

The datasets used and analyzed in this study are available from the corresponding author on reasonable request.

## Abbreviations

MOC: Migrant Older with Children
PSUs: Primary Sampling Units
SSUs: Secondary Sampling Units
ULS-8: Eight-item version of University of California Los Angeles Loneliness Scale
SF-12: Short-Form Health Survey
PCS: Physical Component Summary
MCS: Mental Component Summary
SD: Standard Deviation
